# Time Series Analysis of Sexual Assault Case Characteristics and the 2007–2008 Period of Post-Election Violence in Kenya

**DOI:** 10.1371/journal.pone.0106443

**Published:** 2014-08-29

**Authors:** Michael P. Anastario, Monica Adhiambo Onyango, Joan Nyanyuki, Karen Naimer, Rachel Muthoga, Susannah Sirkin, Kelle Barrick, Martijn van Hasselt, Wilson Aruasa, Cynthia Kibet, Grace Omollo

**Affiliations:** 1 RTI International, Research Triangle Park, North Carolina, United States of America; 2 Boston University School of Public Health, Boston, Massachusetts, United States of America; 3 Physicians for Human Rights, Boston, Massachusetts, United States of America; 4 Moi Teaching and Referral Hospital, Eldoret, Kenya; 5 Nakuru Provincial General Hospital, Nakuru, Kenya; Indiana University and Moi University, United States of America

## Abstract

**Background:**

Following the declaration that President Mwai Kibaki was the winner of the Kenyan presidential election held on December 27, 2007, a period of post-election violence (PEV) took place. In this study, we aimed to identify whether the period of PEV in Kenya was associated with systematic changes in sexual assault case characteristics.

**Methods and Findings:**

Medical records of 1,615 patients diagnosed with sexual assault between 2007 and 2011 at healthcare facilities in Eldoret (n = 569), Naivasha (n = 534), and Nakuru (n = 512) were retrospectively reviewed to examine characteristics of sexual assault cases over time. Time series and linear regression were used to examine temporal variation in case characteristics relative to the period of post-election violence in Kenya. Key informant interviews with healthcare workers at the sites were employed to triangulate findings. The time series of sexual assault case characteristics at these facilities were examined, with a specific focus on the December 2007–February 2008 period of post-election violence. Prais-Winsten estimates indicated that the three-month period of post-election violence was associated with a 22 percentage-point increase in cases where survivors did not know the perpetrator, a 20 percentage-point increase in cases with more than one perpetrator, and a 4 percentage-point increase in cases that had evidence of abdominal injury. The post-election violence period was also associated with an 18 percentage-point increase in survivors waiting >1 month to report to a healthcare facility. Sensitivity analyses confirmed that these characteristics were specific to the post-election violence time period.

**Conclusion:**

These results demonstrate systematic patterns in sexual assault characteristics during the PEV period in Kenya.

## Introduction

Recent armed conflicts have demonstrated the high incidence of sexual violence in modern warfare [1]–[3], which may occur due to opportunistic environmental conditions, strategies to terrorize, expel, or subjugate victims, and/or in settings where captives have been taken [4]. At the population level, alterations in patterns of sexual assault during times of conflict may be indicative of, and/or characterize mass rape.

Medical evidence of sexual assault is likely to vary by injury definition, examiner training and experience, and examination technique [5]. As such, using medical record data to establish a mass rape can be challenging. However, research methods can provide efficient strategies to establish and characterize the occurrence of a mass rape within a jurisdiction [4]. Over time, sexual assaults may exhibit trends or features within a given population. Significant deviations in sexual assault trends may indicate exogenous impacts to the pattern of criminal activity [6]. In the context of conflicts, case characteristics of sexual assaults during mass rapes may logically deviate from non-conflict related assaults.

Following the disputed Kenyan presidential election held on December 27, 2007, there was a period of post-election violence (PEV) that spanned from December 2007 to February 2008. Crimes against humanity – such as murder, deportation or forcible transfer and persecution, rape, and other forms of sexual violence – were alleged to have occurred [7]. Using data derived from medical record reviews of sexual assault cases reporting to three health facilities located in the Rift Valley from 2007–2011, our objective was to identify whether the three-month PEV time period was associated with systematic changes in sexual assault case characteristics using medical records from three healthcare facilities.

## Methods

Quantitative and qualitative methods were used to triangulate the characteristics of sexual assault cases documented during the PEV period at Moi Teaching and Referral Hospital, Nakuru Provincial General Hospital, and Naivasha District Hospital. Quantitative methods included retrospective review and time series analysis of medical records that included a diagnosis of sexual violence at the three facilities between January 2007 and December 2011. Qualitative methods included conducting key informant, semi-structured interviews with healthcare clinicians at each of these facilities. This research was approved by the Institutional Research and Ethics Committee of Moi Teaching and Referral Hospital, the Boston University Medical Center Institutional Review Board (IRB), and the Research Triangle Institute International IRB.

### Medical record review

Medical records for all patients seen at each of the three facilities with a diagnosis of sexual violence from January 2007 to December 2011 were extracted by a study team during March and April, 2013. In order to inform our power analyses, we used a previous study to inform an estimate of the proportions of soft tissue injuries experienced in women who were assaulted by a stranger in comparison to a known assailant, where a between group proportion of 0.1770 was observed [8]. For the purposes of this power analysis, we used the known/unknown status of the assailant to estimate expectedly elevated rates of injuries during a conflict period. With at least 200 medical records in the specific time periods of interest and 400 in the comparison periods, and assuming the proportion in the time period of interest is 0.552 (0.3750 under the null hypothesis) and the proportion in the comparison periods to be 0.3750, the estimated group sample sizes of 200 interest period and 400 comparison period medical records achieved >95% power to detect the 0.1770 difference with a significance level of 0.050, using a test statistic with a two-sided Z test with pooled variance. Although the time series analysis would pool observations by month, we wanted to maximize the stability of estimates that would be “folded” into each month.

Given that, as documentation improved, more records were available in later years, we aimed to oversample the entire population of medical records for 2007 and 2008, with sampling intervals used to select sexual assault case records derived from 2009, 2010, and 2011. Sampling intervals were determined by taking the remaining differences between 600 and the sum of 2007 and 2008 medical records, and creating an interval for years 2009, 2010, and 2011. A structured data collection guide was used to extract, code, and quantify specific case characteristics from the medical record regarding the sexual assault (e.g., more than one perpetrator, specific injuries). Consent was not obtained for use of medical record data – rather, patient records were anonymized and de-identified prior to analysis.

A team of data collectors was trained at each facility to systematically extract and code elements of the patient medical records. Data collection teams were managed and codes were systematically reviewed for quality by a local co-investigator.

As it relates to this study, the standard of care for sexual assault survivors on the day they present to the healthcare facilities studied includes taking a history, physical exam, laboratory investigations, STI prophylaxis, emergency contraception, analgesics, tetanus toxoid, and referral. If possible the health providers encourage survivors to report the case to the police on the same day, if this had not happened by the time survivors come to the hospital. Medical records (Post Rape Care forms) are filled by nurses, clinical officers and physicians.

A PEV assault variable was developed for cases where the documented date of sexual assault fell within the three months during which the PEV took place (December 2007, January 2008, February 2008). Given that data were collapsed by month to create the units of analysis for the time series analyses, this was the greatest degree of specificity possible to capture likely PEV cases. All other sexual assault cases falling outside of this three-month PEV time period were treated as the comparison group. Case characteristics were coded as binary variables, which became percentages ranging from 0 to 1.0 at the month level. Percentages at the month level represent the percentage of all sexual assault cases during a specific time period that exhibited a case characteristic of interest.

### Qualitative interviews

Qualitative in-depth interviews were conducted with clinicians (medical officers, clinical officers and nurses) at the three hospitals using a semi-structured interview guide. A total of 23 clinicians (6 in Eldoret, 10 in Naivasha, and 7 in Nakuru) participated in a 30–120 minute audiotaped interview. The clinicians were either currently working with, or had worked with sexual violence cases between 2007 and 2011. All study participants provided verbal consent to decrease the potential for breach of confidentiality. Verbal consent was documented by the interviewer – this consent procedure was approved by the ethics committees.

### Data analysis

Data were analyzed using STATA 10 statistical software. Data were cleaned to eliminate cases from the analytic dataset that failed logic and consistency checks. To examine trends in case characteristics over time, we collapsed the dataset by month of assault and calculated the percentage of cases for a given month that exhibited a case characteristic of interest. We examined the first 10 autocorrelations for each outcome series, and calculated the Durbin-Watson statistics based on standard least squares regression to examine autocorrelation in the residuals. We used the Prais-Winsten regression estimator to account for potential autocorrelation in the errors and to assess changes in outcomes during the PEV period, controlling for survivor gender and days lagged between assault and presentation. For outcomes in which the first-order autocorrelation was statistically significant, we also estimated an autoregressive model of order 1, or AR(1) model. This model specification contained a one-period lag of the outcome as an independent variable and was adjusted for survivor gender and days lagged between assault and presentation.

In order to further ensure that associations between the PEV period and outcomes were not artifacts of general record keeping during 2007/2008, we conducted a sensitivity analysis using a “dummy” measure to represent the three-month time period immediately preceding the PEV period (November, October, and September 2007) where medical records should be of relatively comparable quality to those of the PEV period. Sensitivity analyses were conducted for significant PEV findings identified in the time series analyses.

Transcripts of audiotapes from qualitative data were checked against the recordings for accuracy. Two researchers conducted inductive content analysis on all transcripts and independently identified codes and broad themes after which they reached a consensus. Nvivo9 computer software was used to organize interview content into sub-categories and broad themes.

## Results

### Descriptive characteristics of the sample (Table 1)

**Figure 1:**
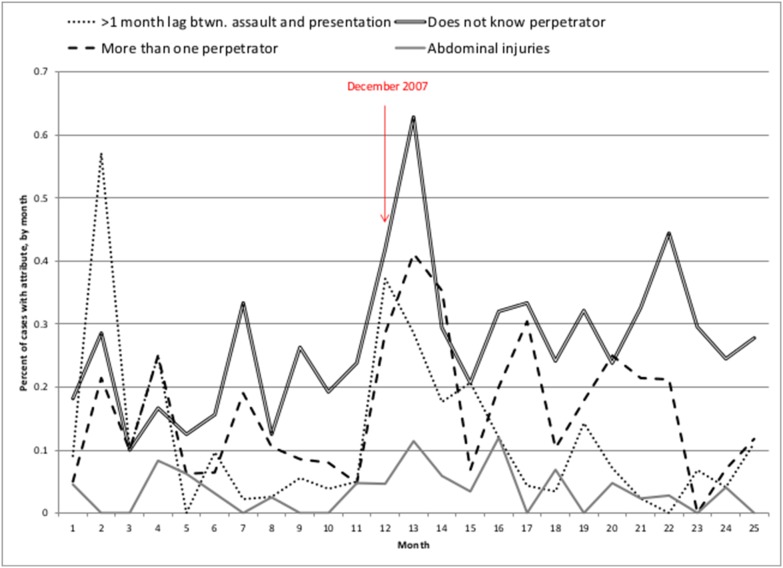
Percent of cases with significant characteristic, by month (January 2007–January 2009)*. *Series has been abbreviated to display multiple characteristics during PEV period.

In total, 1,615 medical records were identified with sexual assault diagnoses, with 569 cases derived from Eldoret, 534 cases from Naivasha, 512 cases from Nakuru. Cases that did not qualify for inclusion failed logic and consistency checks. Cases were collapsed into months for the time series analyses (Table S1 in File S1). Table 1 provides descriptive characteristics of the sample by the mean monthly percentages of the case characteristic for PEV (95 cases, 3 months) and non-PEV (1,520 cases, 57 months) periods. On average, cases in the PEV period showed a greater percentage-point increase in a one-month lag between date of assault and date of presentation to healthcare facility (0.28 in PEV cases, 0.10 in non-PEV cases, p = .003). Cases in the PEV period also showed a greater percentage-point increase in the perpetrator being unknown to the victim (0.45 in PEV cases, 0.23 in non-PEV cases, p = .001), more than one perpetrator being involved in the sexual assault (0.35 in PEV cases, 0.13 in non-PEV cases, p<.001), and abdominal injuries (0.07 in PEV cases, 0.03 in non-PEV cases, p = .025). The monthly time series for case characteristics that were significantly associated with the PEV period are shown in Figure 1. A relative “spike” in these case characteristics is apparent following the December 2007 observation (the first month of the PEV).

**Table 1:** Descriptive Characteristics of the sample, by PEV period.

	Mean monthly prevalencein non-PEV months^a^	Mean monthly prevalencein PEV months^b^	
			P^c^
*Background characteristics*			
Eldoret	0.29	0.57	.631
Naivasha	0.36	0.19	.201
Nakuru	0.35	0.24	.514
Gender (male)	0.07	0.09	.563
**Lag between assault and** **presentation, mean (SD)**	**0.10**	**0.28**	**.003**
*Case characteristics*			
**Did not know perpetrator**	**0.23**	**0.45**	**.001**
**More than 1 perp**	**0.13**	**0.35**	**<.001**
Type of assault:			
Oral	0.01	0.01	.594
Vaginal	0.89	0.86	.550
Anal	0.08	0.11	.364
Weapon used	0.10	0.14	.523
Condom used	0.05	0.03	.524
Rape was witnessed	0.61	0.55	.871
History of previous rape	0.08	0.05	.324
Injury:			
Anogenital	0.70	0.64	.539
Head/neck	0.08	0.06	.472
Limbs	0.08	0.05	.621
Chest	0.02	0.01	.612
Back	0.02	0.02	.719
**Abdomen**	**0.03**	**0.07**	**.025**
Skin	0.02	0.02	.870
Emotional distress	0.25	0.38	.872

a Based on 1,520 cases over 57 non-PEV months.

b Based on 95 cases over 3 PEV months.

c Probability values derived from unadjusted, bivariate Prais-Winsten analyses.

#### Days lagged between assault and presentation to health facility

We analyzed the monthly percentage of cases with more than a one month lag in presentation to a health facility. The first-order autocorrelation was not significant (p = 0.233) and insignificant for two or more lags. The estimated coefficient of the PEV period (beta = 0.18, SE = 0.06) was highly significant (p = 0.003), indicating that the PEV period was associated with an 18 percentage-point increase in cases showing a >1 month lag in presentation to a health facility.

#### Survivor did not know the perpetrator

The first-order autocorrelation was not significant (p = 0.166), and insignificant for two or more lags. After adjusting for sex and lag between date of assault and presentation to the health facility, the estimated coefficient of the PEV period (beta = 0.22, SE = 0.07) was highly significant (p = .002) (Table 2), indicating that the PEV period was associated with a 22 percentage-point increase in cases where the survivor did not know the perpetrator.

**Table 2:** Prais-Winsten models for key outcomes.

Outcome	beta (SE)	P	Durbin-Watson
>1 month lag in presentation^a^	0.18 (0.06)	0.003	1.99
Did not know perpetrator^b^	0.22 (0.07)	0.002	1.99
More than 1 perpetrator^b^	0.20 (0.06)	0.001	1.93
Abdominal injury^b^	0.04 (0.02)	0.048	1.99

a Adjusted for gender only.

b Adjusted for gender and lag between date of assault and presentation to a healthcare facility.

#### Multiple perpetrators for a sexual assault incident

The first-order autocorrelation was marginally significant (p = 0.03) and insignificant for two or more lags. The autocorrelation and partial autocorrelation functions suggested that the percentage of cases where the survivor reported more than one perpetrator did not follow an autoregressive process. After adjusting for sex and lag between date of assault and presentation to the health facility, the Prais-Winsten estimate of the coefficient of the PEV period (beta = 0.20, SE = 0.06) was highly significant (p = 0.001) (Table 2), indicating that the PEV period was associated with a 20 percentage-point increase in cases where the survivor reported more than one perpetrator. We also estimated an AR(1) model, controlling for a one-month lag of y_t_, as a secondary specification. This produced an identical effect size for the PEV period (beta = 0.20, SE = 0.07, p = 0.003).

#### Abdominal injury

The first- and higher-order autocorrelations were not statistically significant. After adjusting for sex and lag between date of assault and presentation to the health facility, the estimated coefficient of the PEV period (beta = 0.04, SE = 0.02) was significant (p = 0.048) (Table 2), indicating that the PEV period was associated with a four percentage-point increase in cases with a documented abdominal injury.

### Sensitivity analyses

No significant differences were observed for cases drawn from the dummy period for the case characteristics that were significantly associated with the PEV period in the Prais-Winsten estimates.

### Qualitative findings

Participants described that during the PEV, few survivors presented to hospital facilities. Lack of knowledge regarding the need to immediately access a health facility, material barriers to accessing a health facility, and the competing responsibility of meeting basic needs (security, shelter, food, childcare) were described as primary barriers to presentation during the PEV period.

Participants described infrastructure issues (blocked roads, violence outside their homes) and general insecurity of the environment as barriers to access during the PEV. One participant described that the delay in presentation:

…was because of stigmatization and, of course, fear, because…who are raping them or defiling the children were people in authority, like the police, the GSU [General Service Unit].

Further, meeting basic material needs was described as taking precedence over survivors reporting to or seeking care at a health facility. As one clinician described:

…they also didn’t have food, they also didn’t have shelter, and most of us were busy taking care of their other needs – like where will they stay, what will they eat? …You’re a mother, with children, and you’re raped, and your house is burnt down. At that time, the priority is: where will your children stay to run for safety. So it does not even occur to you that you have been raped and you may get HIV. So it was a silent emergency, as compared to shelter and food, which were obvious emergencies.

In Eldoret, it was described that following the Waki Commission (the Commission of Inquiry on Post Election Violence) proceedings, survivors of sexual assault began presenting to health facilities. After testifying before the commission, survivors were referred to the clinic.

During the interviews, healthcare workers also described that among the few people who presented at the clinics claiming to have been raped during the PEV period, gang rape was frequently reported. In addition, the majority of the women who presented late with claims of sexual assault were either pregnant or wanted to be tested for the HIV infection.

## Discussion

In sexual assaults at three healthcare facilities, we found that the December 2007–February 2008 PEV period was associated with survivors waiting >1 month to report to a health facility, not knowing the perpetrator, reporting more than one perpetrator, and having documented abdominal injuries. Sensitivity analyses revealed that none of the PEV associations were significantly replicated during the dummy period immediately preceding the PEV. Our findings of systematic alterations in the series of sexual assault cases during the PEV period have implications for establishing the occurrence of mass rape, and characterizing the sexual assaults that took place during the PEV.

This is the first study to use medical record review to illustrate systematic alterations in sexual assault case characteristics during the PEV period. One study conducted at Kenyatta National Hospital examined medical records to establish a relative increase of sexually abused persons treated at the hospital in the year 2007 compared to the years 2006 and 2008 [9]. Our study illustrates change in the months (vs. year) associated with the PEV period, and also shows how case characteristics of sexual assault varied. Conflict-related features of mass rape that have been documented in other conflicts include the use of racial epithets used during attacks in which sexual assaults took place, a relatively high male victimization rate, gang rape, and/or the reported victimization of other individuals [10], [11]. Our findings (particularly concerning gang rape) suggest a change in sexual assault perpetration patterns during the PEV period that is consistent with the documentation of mass rape in conflict settings.

After adjustment, there was more than a 20 percentage-point increase in PEV period cases where the survivor did not know the perpetrator. Research suggests that survivors of sexual assault generally know the perpetrator, and that the more intimate the relationship between a survivor of sexual assault and the perpetrator, the more likely a case is to be processed [12]–[16]. Along with the qualitative evidence of gang rape, our findings illustrate a PEV-specific deviation in the perpetrators of sexual assault during the PEV period. Further, our findings corroborate the testimony of survivors who testified before the Commission of Inquiry on Post-Election Violence (Waki Commission), where the majority reported gang rape during the PEV, including gang rape perpetrated by civilians, security agents, and police [17].

Further, we found that PEV survivors more often waited >1 month following their assault to present to a health facility. This lag period was in part due to barriers of accessibility, security, fear, and lack of community knowledge. Most medical forensic evidence of sexual assault at the patient level could not be readily collected after this type of lag period – particularly viable DNA evidence. However, this lag period itself is a significant finding. Our qualitative findings suggested that several PEV assault survivors sought medical care as a result of pregnancy. A post-hoc analysis revealed that 23.2% of PEV assaults arrived at the clinic seven to nine months following their PEV assault (correspond with the third trimester of pregnancy). In comparison, only 0.4% of non-PEV assaults exhibited this same lag. Even after controlling for this lag, a pattern of case characteristics was still significantly associated with the PEV period. An extended lag period between date of assault and presentation to a medical facility has also been documented in another resource restricted setting affected by armed conflict [18].

One unexpected finding was the increase in the documentation of abdominal injuries associated with the PEV period. There are several possible reasons for why this case characteristic was significantly associated with the PEV period. One possible physiological explanation is that the abdominal injuries documented in the current study were the result of infections derived from the assault. It is also possible that abdominal pain reported was the embodiment of trauma, or the chief presenting complaint of the survivor. In a study of persistent pain in survivors of torture, female abdominal/pelvic/genital pain was associated with sexual assault [19]. Further research is needed to understand the mechanisms underlying the link between abdominal injuries and the PEV period.

### Limitations

Our study has several limitations. First, data are representative of sexual assault cases that were documented in medical records at three facilities within Kenya’s Rift Valley. It is likely that a large number of PEV cases were never reported to a health facility. Further, our findings did not document sexual homicides, only case characteristics of living patients. A strength of our study was using time series analysis in conjunction with medical record review to gain efficiencies that would be lost in conducting a larger, cross-sectional population base survey that rely on survivor recall. Second, the quality of medical records varied over time and by location. Despite our use of a trained data collection team and use of a standardized instrument for extracting case elements from sexual violence files, time-dependent trends in documentation and record keeping may have impacted the rates reported. However, our use of time series analysis and the sensitivity measure (the three months immediately preceding the 2007 PEV) suggests that the variation in the PEV cases represents a true departure from the underlying ‘series’ of sexual assault cases occurring outside of the PEV period. Finally, we were unable to quantitatively capture/report age of victim due to the medical record entry form.

### Conclusion

In this study, which used time series analysis of medical record data to test for systematic changes in sexual assault characteristics for cases presenting to health facilities in the Rift Valley region, we found that cases of assault during the PEV period were associated with an increased percentage of cases where the survivor waited to present to a health facility following the assault, did not know the perpetrator, reported more than one perpetrator, and had evidence of abdominal injury in the medical record. These results illustrate systematic alterations in sexual assault case characteristics during the 2007/2008 period of PEV in Kenya.

## Supporting Information

File S1 **This file contains Table S1 and Figure S1.** Table S1. Time series data for sexual assault case characteristics, 2007–2011. Figure S1. Variables for Table S1.
(PDF)
