# Supplementary material for: Time Series Analysis of Sexual Assault Case Characteristics and the 2007–2008 Period of Post-Election Violence in Kenya
Source: PLoS One. 2014 Aug 29;9(8):e106443. doi: 10.1371/journal.pone.0106443 (PMC4149572; doi:10.1371/journal.pone.0106443)
Supplement: File S1 — This file contains Table S1 and Figure S1. Table S1. Time series data for sexual assault case characteristics, 2007–2011. Figure S1. Variables for Table S1. (PDF) [file pone.0106443.s001.pdf]

Table S1. Time series data for sexual assault case characteristics, 2007-2011

A	B	C	D	E	F	G	H	I	J	K	L	M	N	O	P	Q	R	S	T	U	V
1	0	0	0.182	0.050	0.000	0.955	0.000	0.000	0.000	0.364	0.091	0.000	0.000	0.045	0.000	0.000	0.227	0.045	0.000	0.091	0.000
2	0	0	0.286	0.214	0.000	0.714	0.143	0.143	0.000	0.429	0.071	0.071	0.000	0.000	0.000	0.071	0.571	0.071	0.000	0.571	0.143
3	0	0	0.100	0.100	0.000	1.000	0.100	0.100	0.000	0.400	0.100	0.000	0.000	0.000	0.000	0.300	0.600	0.100	0.000	0.100	0.000
4	0	0	0.167	0.250	0.000	1.000	0.000	0.250	0.000	0.417	0.083	0.000	0.000	0.083	0.000	0.000	0.333	0.083	0.000	0.250	0.000
5	0	0	0.125	0.063	0.000	0.813	0.063	0.188	0.063	0.688	0.063	0.000	0.000	0.063	0.000	0.500	0.813	0.125	0.063	0.000	0.063
6	0	0	0.156	0.065	0.000	0.813	0.000	0.031	0.031	0.406	0.094	0.000	0.000	0.031	0.031	0.094	0.469	0.063	0.125	0.097	0.000
7	0	0	0.333	0.190	0.000	0.867	0.067	0.067	0.000	0.533	0.067	0.000	0.000	0.000	0.000	0.244	0.556	0.044	0.022	0.022	0.044
8	0	0	0.125	0.105	0.000	0.675	0.050	0.125	0.000	0.500	0.050	0.025	0.000	0.025	0.025	0.125	0.625	0.075	0.050	0.026	0.050
9	0	1	0.263	0.086	0.000	0.763	0.079	0.211	0.053	0.500	0.026	0.000	0.079	0.000	0.000	0.132	0.632	0.105	0.026	0.056	0.079
10	0	1	0.192	0.080	0.000	1.000	0.000	0.154	0.077	0.423	0.000	0.000	0.038	0.000	0.000	0.346	0.462	0.115	0.115	0.038	0.000
11	0	1	0.238	0.048	0.000	0.905	0.048	0.190	0.095	0.571	0.048	0.000	0.048	0.048	0.000	0.429	0.857	0.095	0.048	0.050	0.048
12	1	0	0.419	0.286	0.023	0.814	0.140	0.070	0.047	0.628	0.023	0.023	0.047	0.047	0.000	0.233	0.605	0.047	0.047	0.372	0.070
13	1	0	0.629	0.412	0.000	0.886	0.086	0.114	0.057	0.314	0.000	0.000	0.000	0.114	0.000	0.486	0.657	0.029	0.057	0.286	0.086
14	1	0	0.294	0.353	0.000	0.882	0.118	0.235	0.000	0.706	0.118	0.000	0.000	0.059	0.059	0.412	0.647	0.118	0.059	0.176	0.118
15	0	0	0.207	0.069	0.000	0.828	0.103	0.103	0.000	0.690	0.103	0.000	0.034	0.034	0.069	0.483	0.517	0.034	0.103	0.207	0.103
16	0	0	0.320	0.200	0.000	0.920	0.080	0.120	0.080	0.520	0.040	0.000	0.080	0.120	0.080	0.440	0.760	0.160	0.200	0.120	0.040
17	0	0	0.333	0.304	0.000	0.958	0.042	0.167	0.083	0.500	0.083	0.000	0.000	0.000	0.000	0.417	0.708	0.042	0.083	0.043	0.042
18	0	0	0.241	0.103	0.000	0.862	0.034	0.103	0.069	0.690	0.138	0.000	0.034	0.069	0.000	0.552	0.621	0.103	0.103	0.034	0.069
19	0	0	0.321	0.179	0.036	0.750	0.250	0.071	0.036	0.607	0.107	0.000	0.036	0.000	0.000	0.607	0.750	0.107	0.107	0.143	0.214
20	0	0	0.238	0.250	0.024	0.929	0.024	0.095	0.071	0.595	0.095	0.024	0.095	0.048	0.048	0.476	0.786	0.095	0.071	0.071	0.024
21	0	0	0.326	0.214	0.023	0.907	0.093	0.140	0.093	0.651	0.093	0.023	0.023	0.023	0.023	0.256	0.791	0.070	0.023	0.023	0.093
22	0	0	0.444	0.212	0.000	0.889	0.111	0.194	0.083	0.639	0.000	0.056	0.083	0.028	0.028	0.194	0.750	0.167	0.111	0.000	0.083
23	0	0	0.295	0.000	0.000	0.886	0.023	0.023	0.000	0.682	0.045	0.023	0.000	0.000	0.045	0.227	0.727	0.091	0.091	0.068	0.023
24	0	0	0.245	0.070	0.000	0.939	0.041	0.000	0.082	0.531	0.061	0.020	0.020	0.041	0.000	0.469	0.714	0.082	0.204	0.041	0.020
25	0	0	0.278	0.118	0.000	0.833	0.167	0.111	0.111	0.722	0.167	0.000	0.000	0.000	0.056	0.222	0.667	0.167	0.000	0.111	0.056
26	0	0	0.259	0.125	0.000	0.852	0.111	0.111	0.074	0.370	0.037	0.037	0.037	0.037	0.074	0.222	0.556	0.222	0.037	0.074	0.111
27	0	0	0.310	0.200	0.000	0.857	0.000	0.119	0.048	0.524	0.071	0.000	0.071	0.048	0.024	0.190	0.619	0.119	0.214	0.048	0.048
28	0	0	0.400	0.200	0.000	1.000	0.000	0.100	0.000	0.400	0.000	0.000	0.000	0.000	0.050	0.200	0.450	0.050	0.150	0.050	0.000
29	0	0	0.500	0.333	0.000	0.833	0.167	0.333	0.167	0.333	0.000	0.000	0.000	0.000	0.167	0.167	0.500	0.167	0.167	0.167	0.167
30	0	0	0.000	0.091	0.000	0.909	0.091	0.000	0.182	0.455	0.182	0.000	0.000	0.091	0.000	0.091	0.455	0.000	0.000	0.273	0.091
31	0	0	0.188	0.188	0.000	0.875	0.125	0.063	0.000	0.500	0.125	0.000	0.000	0.000	0.063	0.188	0.750	0.188	0.063	0.125	0.188

A	B	C	D	E	F	G	H	I	J	K	L	M	N	O	P	Q	R	S	T	U	V
32	0	0	0.190	0.050	0.000	0.857	0.000	0.000	0.048	0.571	0.048	0.000	0.048	0.000	0.000	0.095	0.524	0.048	0.000	0.000	0.143
33	0	0	0.280	0.160	0.000	0.960	0.000	0.120	0.040	0.720	0.160	0.000	0.040	0.000	0.000	0.480	0.800	0.040	0.040	0.160	0.000
34	0	0	0.167	0.143	0.028	0.889	0.083	0.028	0.111	0.639	0.194	0.056	0.000	0.028	0.028	0.528	0.833	0.111	0.139	0.056	0.111
35	0	0	0.244	0.125	0.000	0.951	0.049	0.098	0.073	0.610	0.098	0.049	0.049	0.049	0.049	0.610	0.878	0.122	0.073	0.049	0.049
36	0	0	0.320	0.200	0.000	0.960	0.040	0.120	0.040	0.520	0.080	0.080	0.000	0.040	0.000	0.520	0.960	0.080	0.040	0.125	0.040
37	0	0	0.200	0.000	0.000	0.960	0.040	0.040	0.000	0.600	0.120	0.000	0.000	0.000	0.000	0.080	0.840	0.000	0.000	0.080	0.080
38	0	0	0.222	0.059	0.000	0.944	0.056	0.056	0.111	0.833	0.056	0.000	0.000	0.056	0.056	0.111	0.889	0.000	0.000	0.111	0.056
39	0	0	0.265	0.030	0.000	0.941	0.059	0.059	0.088	0.706	0.118	0.029	0.000	0.088	0.000	0.206	0.824	0.029	0.088	0.088	0.029
40	0	0	0.045	0.190	0.000	1.000	0.000	0.045	0.000	0.682	0.000	0.000	0.045	0.000	0.000	0.136	0.773	0.045	0.091	0.091	0.000
41	0	0	0.294	0.133	0.000	0.824	0.176	0.294	0.000	0.882	0.118	0.000	0.000	0.118	0.000	0.176	0.941	0.059	0.059	0.176	0.176
42	0	0	0.286	0.111	0.000	0.893	0.107	0.250	0.107	0.714	0.143	0.071	0.036	0.107	0.000	0.107	0.857	0.179	0.036	0.036	0.071
43	0	0	0.250	0.094	0.000	0.906	0.031	0.063	0.094	0.875	0.094	0.000	0.000	0.031	0.000	0.188	0.750	0.094	0.000	0.031	0.063
44	0	0	0.150	0.000	0.000	0.800	0.250	0.000	0.100	0.800	0.000	0.000	0.050	0.000	0.050	0.200	0.700	0.050	0.000	0.150	0.150
45	0	0	0.214	0.037	0.000	0.893	0.036	0.000	0.036	0.607	0.107	0.000	0.036	0.000	0.036	0.214	0.821	0.036	0.071	0.107	0.000
46	0	0	0.100	0.080	0.020	0.800	0.040	0.060	0.100	0.640	0.100	0.120	0.020	0.020	0.040	0.460	0.880	0.100	0.160	0.020	0.060
47	0	0	0.186	0.119	0.000	0.930	0.047	0.000	0.047	0.767	0.093	0.023	0.023	0.023	0.023	0.209	0.791	0.070	0.070	0.023	0.070
48	0	0	0.167	0.111	0.000	0.889	0.167	0.000	0.000	0.667	0.056	0.000	0.000	0.056	0.056	0.167	0.722	0.167	0.111	0.111	0.111
49	0	0	0.333	0.222	0.000	1.000	0.000	0.056	0.000	0.778	0.056	0.000	0.000	0.056	0.000	0.167	0.889	0.056	0.056	0.222	0.000
50	0	0	0.182	0.273	0.000	0.727	0.182	0.182	0.000	0.818	0.000	0.000	0.000	0.000	0.000	0.182	0.636	0.000	0.091	0.182	0.182
51	0	0	0.429	0.143	0.000	1.000	0.000	0.286	0.000	0.571	0.071	0.071	0.071	0.071	0.071	0.143	0.786	0.286	0.286	0.143	0.000
52	0	0	0.118	0.125	0.059	0.882	0.118	0.059	0.118	0.588	0.000	0.000	0.000	0.000	0.000	0.118	0.706	0.059	0.059	0.118	0.059
53	0	0	0.182	0.091	0.091	0.909	0.091	0.000	0.000	0.818	0.091	0.000	0.000	0.000	0.000	0.091	0.636	0.000	0.000	0.000	0.091
54	0	0	0.136	0.095	0.000	0.636	0.409	0.091	0.045	0.727	0.045	0.045	0.000	0.000	0.000	0.182	0.682	0.045	0.045	0.318	0.318
55	0	0	0.000	0.000	0.000	0.938	0.063	0.000	0.000	0.563	0.188	0.000	0.000	0.000	0.000	0.063	0.813	0.000	0.000	0.125	0.063
56	0	0	0.056	0.056	0.000	0.889	0.056	0.000	0.111	0.722	0.111	0.000	0.000	0.000	0.000	0.111	0.889	0.111	0.167	0.056	0.056
57	0	0	0.355	0.129	0.000	0.935	0.065	0.258	0.000	0.645	0.129	0.000	0.065	0.097	0.000	0.161	0.710	0.032	0.097	0.000	0.065
58	0	0	0.208	0.064	0.000	0.917	0.104	0.146	0.083	0.625	0.083	0.000	0.000	0.021	0.042	0.146	0.792	0.083	0.000	0.022	0.083
59	0	0	0.200	0.158	0.025	0.925	0.025	0.075	0.075	0.600	0.125	0.050	0.075	0.075	0.075	0.375	0.800	0.125	0.250	0.025	0.050
60	0	0	0.231	0.269	0.000	0.808	0.115	0.038	0.115	0.731	0.154	0.000	0.000	0.000	0.000	0.462	0.731	0.000	0.077	0.000	0.115

Figure S1. Variables for Table S1

Field	Description
A	Month
B	Month of post-election violence
C	Sensitivity measure for dummy analysis
D	Survivor did not know perpetrator
E	More than one perpetrator
F	Oral assault
G	Vaginal assault
H	Anal assault
I	Weapon used
J	Condom used
K	Rape was witnessed
L	History of previous rape
M	Chest injury
N	Back injury
O	Abdominal injury
P	Skin injury
Q	Emotional distress
R	Anogenital injury
S	Head/neck injury
T	Limbs injury
U	> month lag between date of assault and date of presentation to healthcare facility
V	Male
